# Burden of female breast cancer in India: estimates of YLDs, YLLs, and DALYs at national and subnational levels based on the national cancer registry programme

**DOI:** 10.1007/s10549-024-07264-3

**Published:** 2024-03-04

**Authors:** Vaitheeswaran Kulothungan, Thilagavathi Ramamoorthy, Krishnan Sathishkumar, Rohith Mohan, Nifty Tomy, G. J. Miller, Prashant Mathur

**Affiliations:** Indian Council of Medical Research (ICMR) – National Centre for Disease Informatics and Research (NCDIR), Nirmal Bhawan – ICMR Complex (II Floor), Poojanahalli, Kannamangala Post, Bengaluru, Karnataka 562 110 India

**Keywords:** Breast cancer, Cancer, Cancer burden, DALYs, India

## Abstract

**Purpose:**

Female breast cancer (BC) is the leading cause of cancer incidence and mortality in India, and accounted for 13.5% of new cancer cases and 10% of cancer-related deaths in 2020. This study aims to estimate and report the female BC burden in India at state level from 2012 to 2016 in terms of years of life lost, years lived with disability, and disability-adjusted life years (DALYs), and to project the burden for the year 2025.

**Methods:**

The cancer incidence and mortality data from 28 population-based cancer registries were analysed. The mean mortality to incidence ratio was estimated, and mortality figures were adjusted for underreporting. The burden of female BC was estimated at national and subnational levels using Census data, World Health Organisation’s lifetables, disability weights, and the DisMod-II tool. A negative binomial regression is employed to project burden for 2025.

**Results:**

The burden of BC among Indian women in 2016 was estimated to be 515.4 DALYs per 100,000 women after age standardization. The burden metrics at state level exhibited substantial heterogeneity. Notably, Tamil Nadu, Telangana, Karnataka, and Delhi had a higher burden of BC than states in the eastern and north-eastern regions. The projection for 2025 indicates to a substantial increase, reaching 5.6 million DALYs.

**Conclusion:**

The female BC burden in India was significantly high in 2016 and is expected to substantially increase. Undertaking a multidisciplinary, context-specific approach for its prevention and control can address this rising burden.

**Supplementary Information:**

The online version contains supplementary material available at 10.1007/s10549-024-07264-3.

## Introduction

Cancer is estimated to have caused 9·6 million global deaths in 2017 [1]. Globally, cardiovascular diseases are the leading cause of disability-adjusted life years (DALYs), followed by cancer. Although nearly half of all cancer cases occur in countries with a high Sustainable Development Index (SDI), only a quarter of the DALYs burden is borne by these countries [1]. This suggests that countries with a low SDI face a significant cancer burden. For India, Global cancer observatory (GLOBOCAN) projected 1.3 million new cancer cases and approximately 850,000 cancer-related deaths by 2020. Breast cancer was the leading cause of cancer incidence and mortality in India, accounting for 13.5% of new cancer cases and 10.6% of all cancer deaths [2].

In addition, there is a disparity in leveraging the accessibility of existing facilities and the detection of disease in its advanced stages, putting the survival of women with breast cancer at risk despite advancements in treatment modalities. Patients with stage I disease had a better survival rate of 93.3%, while those with stage IV disease had a survival rate of 24.5%, with an overall survival rate of 73.8% [3]. A population-based study conducted in South India indicated that patients with a lower educational background have lower survival rates which can be attributed to their cancer being in a more advanced stage at the time of diagnosis [4].

Population-based cancer registries (PBCRs) provide a solid foundation for studying the cancer burden across regions and over time in India. Multiple cancer burden studies, including the most recent study by the Indian Council of Medical Research-National Centre of Disease Informatics and Research (ICMR-NCDIR) on cancer burdens in India, have relied on these data [5]. Breast cancer accounted for 21.8% of the total cancer burden in women as measured by DALYs. Under the National Programme for Noncommunicable Diseases (NP-NCD), the Ministry of Health and Family Welfare (MoHFW) has implemented breast cancer population screening programme for all women aged between 30 and 69 years [6]. Despite this, only 1.6% of women aged 30–69 years in India have ever undergone breast cancer screening ﻿[7].

India is not a homogeneous country, and each state has distinct demographic, economic, and cultural characteristics. In addition, it has been observed that the incidence, mortality, and mortality-to-incidence ratio (MIR) of breast cancer are closely related to the geographical indicators of development [8]. Examining breast cancer epidemiology at regional levels could, therefore, aid policymakers in tailoring screening and treatment programmes based on local needs to effectively combat the growing breast cancer burden. This study aims to estimate and report the state-wise burden of female breast cancer in India from 2012 to 2016 in terms of Years Lived with Disability (YLDs), Years of Life Lost (YLLs) and DALYs, and to project the burden for the year 2025.

## Materials and methods

Population-based cancer registries (PBCRs) serves as reliable and consistent, long-term, national sources of data on the incidence, survival estimates, and trends of cancer in the country. Since the establishment of the National Cancer Registry Programme (NCRP) in 1981, it has served as a valuable data source on the burden of cancer through its network of 28 PBCRs that covers 3.5% of rural, 42.9% of urban, and 53.6% of semi-urban population [9–11].

Breast cancer incidence and mortality rates for women of various ages were obtained from 28 different PBCRs across India. Breast cancer incidence and mortality were determined using the ICD-10 code “C50” [12]. We searched for available evidence on the reported mortality to incidence ratio (MIR) in India to adjust the mortality numbers for underreporting of mortality in the country. Two national level studies were identified, with an average MIR for cancer of 35.0% and 75.4% [13, 14]. As a result, all longitudinal data points with reported MIR of 35.0% for breast cancer from PBCRs between 2005 and 2016 were extracted to estimate a standard MIR for breast cancer among Indian women. A total of 33 data points had an MIR of 35.0%, from which a mean MIR of 45.7 was estimated for breast cancer. Based on Akaike's and Bayesian information criteria, the gamma distribution was identified as the best fit to the data. The mean MIR was calculated using the Markov Chain Monte Carlo method. For the registries with reported MIRs less than 45.7%, the MIR was replaced with 45.7%, and adjusted mortality numbers were computed. These adjusted mortality figures were used for further analysis and burden estimation.

Breast cancer incidence and mortality rates were calculated for quinquennial age groups. The incidence rate for a specific group was calculated by dividing the number of newly diagnosed breast cancer cases in a given year by the corresponding mid-year population. Similarly, the mortality rate for a specific group was calculated by dividing the number of breast cancer deaths in a given year (adjusted) by the corresponding mid-year population. The difference distribution method was used to project state-level populations by age for the years 2012 to 2016. The estimated age wise distribution of the female population at all PBCRs for the years 2012 to 2016 is given in Supplementary Table 1.

### Estimation of burden

The breast cancer burden was estimated and reported in terms of YLLs, YLDs, and DALYs per 100,000 female population for each state using the corresponding available registry. If registry data for a specific state were unavailable, the closest possible registry data were used for estimation. YLLs were calculated by multiplying the total number of breast cancer deaths in a given age group by the standard life expectancy for that age group. The WHO standard life tables were used to calculate the standard life expectancies of different age groups (Supplementary Table 2). YLDs were calculated by multiplying the total number of prevalent breast cancer cases by the disability weight. The prevalence estimates were generated using the DisMod-II tool [15] with incidence, mortality, MIR, total population, and all-cause mortality rate as inputs. The Sample Registration System (SRS) was used to obtain age-specific all-cause mortality rates for women between 2012 and 2016 [16]. According to the Global Burden of Disease study, the disability weight for metastatic cancer is 0.45 [17]. The DALY metrics were computed by adding the estimated YLLs and YLDs. Finally, the burden metrics (YLLs, YLDs, and DALYs) were age standardized using the WHO World Population Standard Distribution [18]. Furthermore, breast cancer burden metrics for 2025 were projected using data from 2001 to 2016. Negative binomial regression was used for projection because the conditional mean of the burden metrics was less than the conditional variance.

## Results

### Breast cancer burden at regional level

**Fig. 1:**
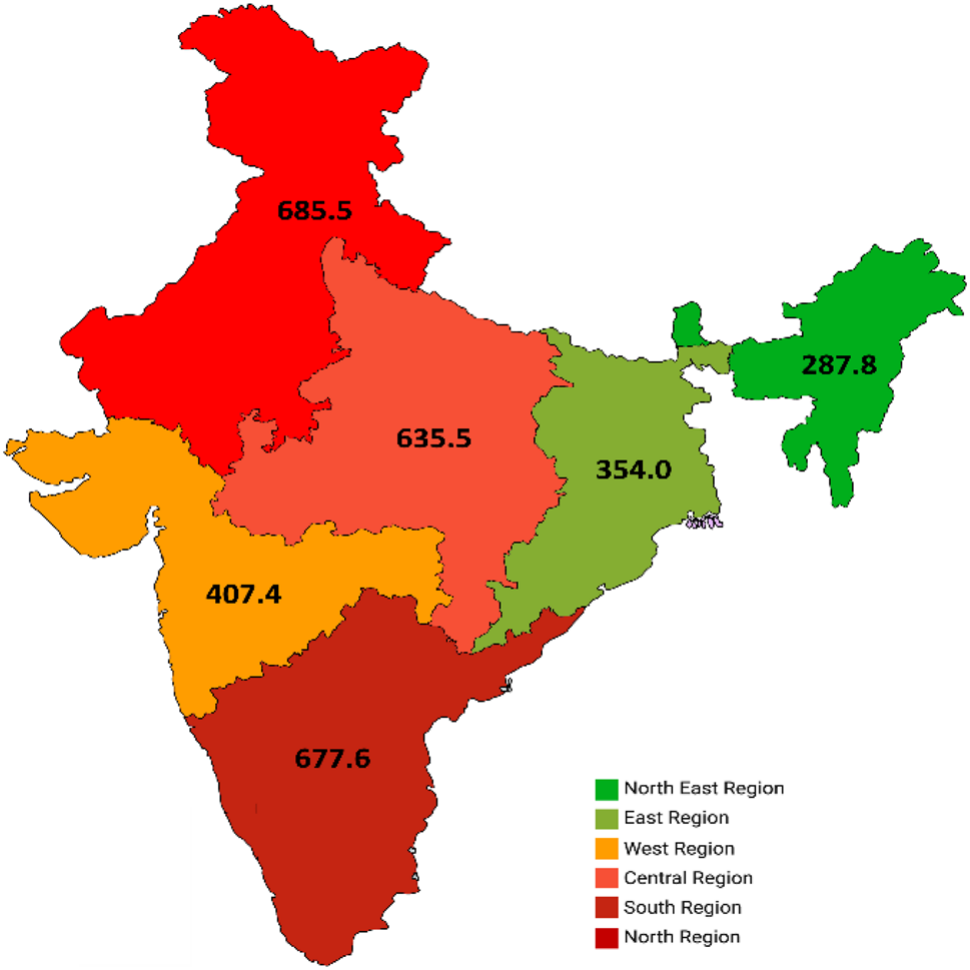
Distribution of age standardized DALYs–ASR per 100,000 women for breast cancer in 2016 by Region

Breast cancer was estimated to result in 515.4 Age-Standardized Rate (ASR)-DALYs per 100,000 Indian women in 2016. Of this, only 14.2 DALYs were contributed by YLDs, while the remaining were contributed by YLLs. The overall burden before age standardization was 468.2 DALYs per 100,000 women. Notably, the burden varied significantly by region. The northern and southern regions exhibited the highest burden in the country, with 685.5 and 677.6 DALYs per 100,000 women, respectively. Central India followed closely with a breast cancer burden of 635.5 DALYs per 100,000 women, while other parts of the country depicted a lower burden. The north-eastern region had the lowest breast cancer burden, with 287.8 DALYs per 100,000 women (refer to Table 1 and Fig. 1).

**Table 1:** Region- and state-wise burden of female breast cancer (YLLs, YLDs, DALYs) per 100,000 in India for 2016

Regions/States of India	YLLs		YLDs		DALYs		Registries used for burden estimation
	CR	ASR	CR	ASR	CR	ASR	
**North**	**600.7**	**665.8**	**17.6**	**19.7**	**618.3**	**685.5**	
Punjab	618.5	659.4	19.8	20.3	638.4	679.7	Patiala
Jammu and Kashmir	600.7	665.8	17.6	19.7	618.3	685.5	Patiala, Delhi
Haryana	600.7	665.8	17.6	19.7	618.3	685.5	Patiala, Delhi
Himachal Pradesh	600.7	665.8	17.6	19.7	618.3	685.5	Patiala, Delhi
Uttarakhand	600.7	665.8	17.6	19.7	618.3	685.5	Patiala, Delhi
Delhi	582.9	672.2	15.3	19.1	598.2	691.3	Delhi
**South**	**660.8**	**657.9**	**19.1**	**19.7**	**679.9**	**677.6**	
Karnataka	567.9	691.4	16.8	20.6	584.7	712.1	Bangalore
Telangana	615.3	754.3	18.3	24.7	633.6	779.0	Hyderabad District
Kerala	714.4	581.4	19.5	15.9	733.9	597.3	Kollam District, Thiruvananthapuram District
Tamil Nadu	692.1	681.0	21.3	21.2	713.4	702.2	Chennai
Andhra Pradesh	660.8	657.9	19.1	19.7	679.9	677.6	Bangalore, Hyderabad District, Kollam District, Thiruvananthapuram District,Chennai
**East**	**383.7**	**343.2**	**12.1**	**10.9**	**395.8**	**354.0**	
Odisha	383.7	343.2	12.1	10.9	395.8	354.0	Kolkata
Bihar	383.7	343.2	12.1	10.9	395.8	354.0	Kolkata
West Bengal	383.7	343.2	12.1	10.9	395.8	354.0	Kolkata
Jharkhand	383.7	343.2	12.1	10.9	395.8	354.0	Kolkata
**West**	**374.0**	**395.4**	**11.1**	**11.9**	**385.1**	**407.4**	
Gujarat	413.3	423.4	11.2	12.2	424.5	435.6	Ahmedabad Urban
Goa	374.0	395.4	11.1	11.9	385.1	407.4	Mumbai, Barshi rural,Osmanabad & Beed (Barshi Expanded), Pune, Aurangabad, Wardha, Nagpur, Ahmedabad urban
Rajasthan	374.0	395.4	11.1	11.9	385.1	407.4	Mumbai, Barshi rural, Osmanabad & Beed (Barshi Expanded), Pune, Aurangabad, Wardha, Nagpur, Ahmedabad urban
Maharashtra	368.4	391.4	11.0	11.9	379.4	403.3	Mumbai, Barshi rural,Osmanabad & Beed (Barshi Expanded), Pune, Aurangabad, Wardha, Nagpur
**Central**	**525.5**	**619.1**	**13.2**	**16.4**	**538.6**	**635.5**	
Uttar Pradesh	525.5	619.1	13.2	16.4	538.6	635.5	Bhopal
Madhya Pradesh	525.5	619.1	13.2	16.4	538.6	635.5	Bhopal
Chhattisgarh	525.5	619.1	13.2	16.4	538.6	635.5	Bhopal
**Northeast**	**220.9**	**275.1**	**9.3**	**12.8**	**230.2**	**287.8**	
Manipur	146.4	183.9	4.1	5.0	150.6	188.9	Manipur
Mizoram	291.6	395.7	8.9	11.7	300.5	407.5	Mizoram
Sikkim	192.0	215.5	5.6	7.1	197.5	222.6	Sikkim
Tripura	148.1	163.5	3.5	4.0	151.6	167.5	Tripura
Meghalaya	84.4	138.1	2.6	4.3	87.0	142.4	Meghalaya
Nagaland	136.0	200.7	5.0	9.4	141.0	210.1	Nagaland
Arunachal Pradesh	228.8	320.9	19.0	29.4	247.8	350.3	West Arunachal (Naharlagun), Pasighat
Assam	324.6	362.2	11.6	13.4	336.2	375.6	Dibrugarh District, Kamrup Urban, Cachar District
**India**	**455.7**	**501.2**	**12.6**	**14.2**	**468.2**	**515.4**	

Bold represents national and regional estimations

Abbreviations: CR–Crude rate; ASR–Age-standardized rate, YLL–Years of Life lost, YLD–years lived with disability, DALYs–Disability-adjusted life years

### Breast cancer burden state-wise

**Fig. 2:**
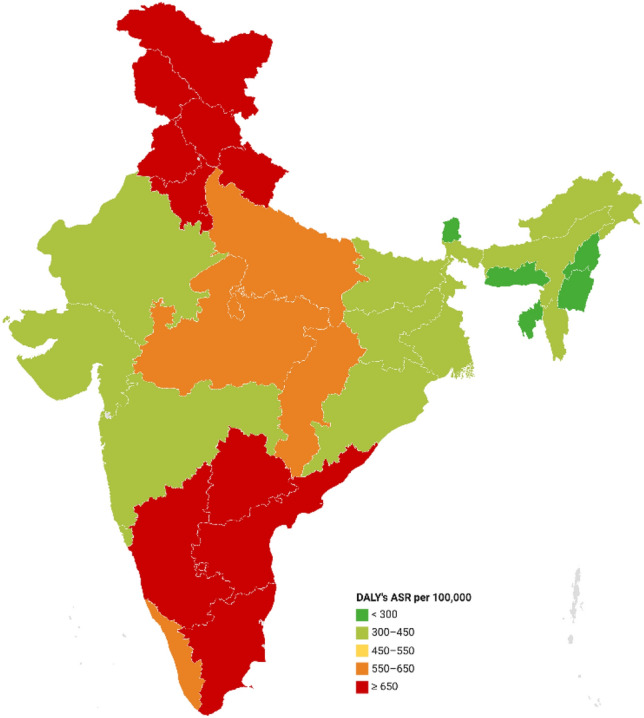
Distribution of age standardized DALYs–ASR per 100,000 women for breast cancer in 2016 by State

At the state level, Kerala had the highest crude rate of DALYs in India, with 733.9 per 100,000 women, followed by Tamil Nadu. Meghalaya exhibited a remarkably low burden at 87 DALYs per 100,000 women. However, on age standardization, the rankings of the states changed due to different age structures. Three southern states, Telangana, Karnataka, and Tamil Nadu, had a breast cancer burden of more than 700 DALYs per 100,000 women, with Telangana having the highest (779.0 DALYs per 100,000 women). The age-adjusted female breast cancer burden in Kerala dropped to 597.3 DALYs per 100,000 women. Several northern and southern states also had high burdens, with 600 DALYs per 100,000 women. Many north-eastern states had a lower burden of breast cancer, with Meghalaya having the lowest burden (142.4 DALYs per 100,000 women) (refer to Table 1 and Fig. 2).

### Proportion contribution of YLLs and YLDs

**Fig. 3:**
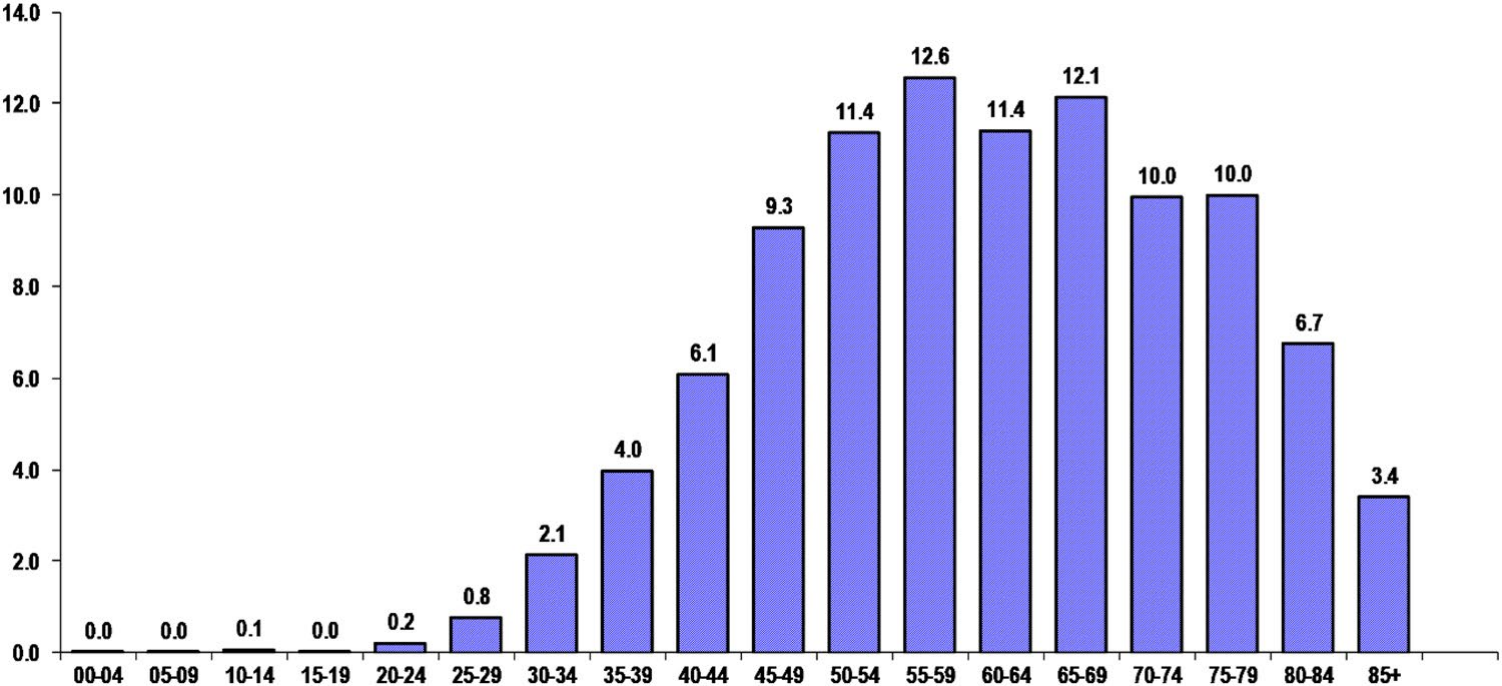
Age wise percentage distribution of DALYs per 100,000 women for breast cancer in India during 2016

An analysis of the burden in terms of YLLs and YLDs revealed a similar pattern. The burden of YLLs and YLDs was highest in the northern, southern, and central regions of India, while it was lowest in the western, eastern, and north-eastern regions. Arunachal Pradesh was an exception, with a significantly high burden of disability due to breast cancer (YLDs: 29.4 per 100,000 women). The age distribution of DALYs (refer to Fig. 3) indicated that women aged 50 to 69 years experienced the highest burden of breast cancer. The burden gradually increased to this maximum and then gradually decreased after 69 years, with more than three quarters of the breast cancer burden occurring after the reproductive age group of 49 years.

Table 2 displays the crude as well as age-standardized incidence and mortality rates for each registry. The crude incidence rate was highest in the Thiruvananthapuram district registry (47.0 per 100,000 women), followed by Chennai and Kollam districts. However, on age standardization, urban-based registries such as Chennai, Bangalore, Hyderabad district, and Delhi had the highest incidence of breast cancer, whereas mostly rural registries like those in the north-eastern states and Barshi rural had very low incidence rates. This urban–rural split was also observed in age-standardized mortality rates. The national age-standardized rates of breast cancer incidence and mortality were 32.0 and 15.1 per 100,000 women, respectively.

**Table 2:** Registry wise burden of female breast cancer (Incidence, Mortality, YLLs, YLDs, DALYs) per 100,000 in India for 2016

Registries	Incidence		Mortality		YLLs		YLDs		DALYs	
	CR	ASR	CR	ASR	CR	ASR	CR	ASR	CR	ASR
Bangalore	36.1	45.2	16.5	21.7	567.9	691.4	16.8	20.6	584.7	712.1
Mumbai	34.9	37.1	16.0	17.5	501.0	518.9	15.6	16.6	516.6	535.5
Chennai	46.0	46.0	21.0	21.7	692.1	681.0	21.3	21.2	713.4	702.2
Hyderabad District	34.3	46.6	15.7	21.2	615.3	754.3	18.3	24.7	633.6	779.0
Bhopal	27.9	34.9	12.7	16.0	525.5	619.1	13.2	16.4	538.6	635.5
Delhi	32.9	41.2	15.1	18.9	582.9	672.2	15.3	19.1	598.2	691.3
Barshi Rural	13.1	13.0	6.5	6.5	217.6	226.1	7.0	6.9	224.6	233.0
Kollam District	40.3	32.7	18.4	14.9	670.8	547.8	18.2	14.8	689.1	562.6
Aurangabad	21.1	27.2	9.7	13.5	324.9	415.9	10.9	14.0	335.8	429.9
Nagpur	28.2	28.5	12.9	13.3	509.4	494.8	15.7	15.5	525.1	510.3
Pune	27.0	32.6	12.4	15.5	412.6	480.8	11.8	14.2	424.4	495.0
Thiruvananthapuram District	47.0	38.2	21.5	17.4	758.0	615.0	20.8	16.9	778.7	631.9
Kolkata	24.9	22.4	11.4	10.4	383.7	343.2	12.1	10.9	395.8	354.0
Dibrugarh District	13.4	15.9	6.1	7.3	266.4	298.4	9.2	11.7	275.6	310.0
Kamrup Urban	26.4	29.5	12.1	13.8	487.3	504.4	13.1	14.6	500.4	519.0
Cachar District	12.3	15.4	5.6	8.0	220.0	283.8	12.5	13.9	232.5	297.7
Manipur State	8.9	10.9	4.1	5.6	146.4	183.9	4.1	5.0	150.6	188.9
Mizoram State	17.3	23.2	7.9	11.5	291.6	395.7	8.9	11.7	300.5	407.5
Sikkim State	9.2	11.6	4.2	5.1	192.0	215.5	5.6	7.1	197.5	222.6
Ahmedabad Urban	23.3	25.5	10.6	11.5	413.3	423.4	11.2	12.2	424.5	435.6
Wardha District	22.5	21.5	10.3	9.9	404.0	387.0	10.4	10.0	414.5	397.0
Tripura State	7.5	8.5	3.4	4.0	148.1	163.5	3.5	4.0	151.6	167.5
Nagaland	6.9	10.0	3.1	5.3	136.0	200.7	5.0	9.4	141.0	210.1
Meghalaya	4.5	7.7	2.0	3.6	84.4	138.1	2.6	4.3	87.0	142.4
West Arunachal	6.8	11.2	3.1	8.0	119.7	244.5	6.9	9.5	126.5	254.1
Osmanabad & Beed	12.3	12.6	5.6	5.6	209.2	216.6	5.8	6.0	215.0	222.6
Pasighat	14.8	19.4	6.8	8.6	338.0	397.2	31.1	49.3	369.1	446.4
Patiala District	38.4	39.8	17.5	19.4	618.5	659.4	19.8	20.3	638.4	679.7
**India**	**28.4**	**32.0**	**13.0**	**15.1**	**455.7**	**501.2**	**12.6**	**14.2**	**468.2**	**515.4**

Bold represents national estimations

Abbreviations: CR–Crude rate; ASR–Age-Standardized rate, YLL–Years of life lost, YLD–Years lived with disability, DALYs–Disability-adjusted life years

### Burden projected for 2025

According to the projections, the burden of female breast cancer in India in 2025 is expected to be 5.6 million DALYs. Premature death due to breast cancer (YLLs) would contribute 5.3 million DALYs to the total burden, with the remaining due to disability (YLDs) (refer to Table 3).

**Table 3:** Projection estimates of YLLs, YLDs, DALYs for female breast cancer in India for 2021 and 2025

Cancer burden metrics	2021	2025
YLLs	4,621,600	5,295,053
YLDs	225,892	258,620
DALYs	4,847,492	5,553,673

## Discussion

This study examines the state-wise burden of female breast cancer in India in 2016 using data from 28 population-based cancer registries under NCRP across the country. In 2018, the age-standardized breast cancer incidence among women in South Central Asia was 25.9 per 100,000 women, according to the GLOBOCAN study [8, 19]. According to the Global Burden of Diseases (GBD) study, the age-standardized rate in South Central Asia in 2016 was 21.6 per 100,000 women [20]. These studies estimated the national and subnational burdens using a wide range of data sources. However, our study only used data from population-based cancer registries under NCRP, which are mainly in urban areas. Rural women are less likely to develop breast cancer than their urban counterparts, and age-standardized incidence rates are higher in urban and metro areas, with Hyderabad, Chennai, Bangalore, and Delhi as leading Indian cities [21, 22]. Urban factors such as a sedentary lifestyle, high obesity rates, delayed age at marriage and childbirth, and minimal breastfeeding have been attributed to a higher burden of breast cancer in urban areas compared to their rural counterparts [23]. This is supported by our study's findings, which indicate that urban registries such as Chennai, Bangalore, and Delhi had higher incidence rates than rural registries. This can explain why our study found a higher incidence of breast cancer (32.0 per 100,000 women) than the GBD and GLOBOCAN estimates.

The study found that the breast cancer burden is higher in the state of Telangana. The relatively low literacy rate among females, higher life expectancy than the national average, high tobacco use, and alcohol consumption among women contributed to the increase in cancer burden in the state [24, 25]. In addition, one in three women were found to be overweight/obese, ranking 6th among the states of India, which is an important risk factor for breast cancer [26]. This was attributed to changing lifestyles, sedentary behaviour, unhealthy eating, and inadequate physical activity [27]. An average delay of 271 days (as noted in a study from neighboring state of Odisha) in disclosing symptoms to loved ones before taking steps toward diagnosis contributes to the late diagnosis of cancer [28].

The GBD also looked at regional variations in female breast cancer. The crude DALYs were found to be higher in states such as Kerala, Punjab, Tamil Nadu, Delhi, Maharashtra, Karnataka, and Haryana [20]. With the exception of Maharashtra, these states have higher DALYs in our study as well. However, the female breast cancer burden estimate for Maharashtra was simply an average of a number of registries that included both urban and rural registries. In our study, we may have underestimated the burden for Maharashtra. However, the majority of these burden differences between states can be explained by their socio-economic development.

Socio-economic factors significantly shape the cancer burden, affecting access to healthcare, preventive measures, and treatment outcomes. Individuals with lower socio-economic status encounter barriers to timely and quality healthcare, leading to delayed cancer detection, compounded by limited resources and health literacy. Occupational exposures and financial strain heighten cancer risks and impact treatment accessibility. Geographical and psychosocial disparities further complicate the issue. Research priorities may inadvertently overlook cancers prevalent in lower socio-economic groups. Recognizing and addressing these disparities is crucial for equitable cancer control, ensuring universal access to prevention, early detection, and treatment. In India, the correlation between cancer prevalence and socio-economic inequalities is evident, emphasizing the need to reevaluate resource allocation and enhance access to healthcare and social support systems [29–32].

Even global studies have found that developed countries have a higher incidence of breast cancer than developing and underdeveloped countries [19]. This can be applied to India, where more developed states like Tamil Nadu, Telangana, Karnataka, and Delhi have a much higher burden of breast cancer than states in the eastern and north-eastern regions. This is due to known risk factors such as delayed first childbirth, lower parity, higher levels of obesity, a shorter duration of breastfeeding, and physical inactivity, all of which are linked to a region's socio-economic development [33]. Another possible explanation is that states with more advanced healthcare infrastructure have higher levels of awareness, screening, and diagnosis rates.

In developing Asian countries, the incidence of breast cancer peaked in the forties during the early twenty-first century, whereas it peaked in the sixties in developed countries [34, 35]. Our finding that breast cancer incidence peaked after the age of 50 suggests that the age of onset in India has changed since the previous decade, moving from the forties to fifty and above. This is a positive finding despite the rising incidence of breast cancer, as the prognosis for breast cancer in younger women is typically worse [36]. The results of the projection indicated a significant increase in the burden of female breast cancer in India from 2016 levels. Several studies in India have found that the age-standardized incidence of breast cancer is significantly increasing [36–38].

The increasing incidence of breast cancer in India underscores the urgent need for comprehensive awareness campaigns and screening programs [39]. A significant concern is that a majority of women diagnosed with breast cancer in the country present with advanced stages or metastatic disease, suggesting a lack of awareness [36]. India faces remarkably low rates of breast cancer screening, encompassing self-breast examination and mammography [40]. Numerous barriers contribute to this, including personal factors (lack of awareness about screening services, methods, lack of prioritization of health, and inadequate education), economic constraints, social stigma around the disease, distrust in the healthcare systems and professionals, inadequate health infrastructure, fears regarding surgical procedures like mastectomy [31, 41–45].

Recognizing the need for intervention, mandatory screening, incentivization, and awareness creation emerge as crucial factors facilitating breast cancer screening. Hence, adopting a multidisciplinary approach that not only raises awareness but also promotes screening and facilitates treatment becomes imperative. Strengthening screening, diagnostic, and treatment facilities for breast cancer patients in India could potentially reduce premature mortality, prevent catastrophic health expenditures, and enhance overall survival rates.

## Strengths and limitations

This study reports the breast cancer burden for each Indian state in terms of YLDs, YLLs, and DALYs. Cancer registries play a vital role in collecting incidence, and mortality data and monitoring and evaluating cancer control programs [46]. The data for the study were collected from 28 population-based cancer registries covering 10% of the population in India's various states with annual coverage ranging from a low of 0.69 lakhs to a high coverage of 81.0 lakhs [22]. Population-based cancer registries also ensure that nearly all incident cases within the target populations are recorded. In India, population-based cancer registries (PBCRs) serve as the primary and reliable long-term data sources, offering valuable insights into the magnitude and patterns of cancer. Due to the limited availability of mortality data, we depend on real-time information from PBCRs for both cancer incidence and mortality statistics. The data from some of the PBCRs are regularly published in successive volumes of Cancer Incidence in Five Continents (CI-5) by WHO-IACR/IARC.

In low- and middle-income countries, only a fraction of deaths are recorded in vital registration systems, which may underestimate cause-specific mortality. In this study, however, this issue has been effectively addressed by calculating the national average MIR and adjusting the mortality numbers to account for this under registration of deaths. This study's estimated MIR was nearly comparable to other MIR estimates for India. GLOBOCAN—2018 estimated India's MIR to be between 0 and 59 [8], while GBD—2016 estimated it to be between 0 and 52 [47].

The location of the functioning cancer registries is an important limitation of this study. Assuming that states in the same region would have similar breast cancer epidemiology, estimates for states without a registry were derived from the registry closest to them. Furthermore, the rural component of PBCRs is not presented in the majority of States. However, healthcare in India is primarily a state subject; consequently, screening rates and treatment efficacy can differ between states within the same region. The socio-economic development of the states within a region can also vary, resulting in variations in the health-promoting behaviours of the populations. Even within states, registries are predominantly urban, limiting their applicability to the entire state.

## Conclusion

In India, the burden metrics for female breast cancer at the state level demonstrate substantial heterogeneity. This is a result of regional and state-specific differences in lifestyle, exposure to risk factors, and healthcare accessibility. These results show how important it is to design and use strategies based on local needs if we want to halt the rising number of breast cancer cases in the country.

### Supplementary Information

Below is the link to the electronic supplementary material.

Supplementary file1 (PDF 115 kb)

## Data Availability

Data is available within this paper. The corresponding author can be contacted at director-ncdir@icmr.gov.in for further clarification if required.

## Abbreviations

BC: Breast cancer
YLLs: Years of life lost
YLDs: Years lived with disability
DALYs: Disability adjusted life years
SDI: Sustainable development index
PBCR: Population-based cancer registries
NP-NCD: National programme for noncommunicable diseases
MoHFW: Ministry of health and family welfare
MIR: Mortality-to-incidence ratio
NCRP: National cancer registry programme
SRS: Sample registration system
WHO: World health organization
ASR: Age standardized rate
GBD: Global burden of diseases
GLOBOCAN: Global cancer observatory
