# Supplementary material for: Burden of female breast cancer in India: estimates of YLDs, YLLs, and DALYs at national and subnational levels based on the national cancer registry programme
Source: Breast Cancer Res Treat. 2024 Mar 4;205(2):323–32. doi: 10.1007/s10549-024-07264-3 (PMC11101532; doi:10.1007/s10549-024-07264-3)
Supplement: Supplementary file 1 — Supplementary file1 (PDF 115 kb) [file 10549_2024_7264_MOESM1_ESM.pdf]

Title: The burden of female breast cancer in India: estimates of YLDs, YLLs, and DALYs at national and subnational levels based on the National Cancer Registry Programme.

Short title: Female Breast Cancer Burden in India for 2025.

Journal Name: Brest Cancer Research and Treatment.

Authors:

Vaitheeswaran Kulothungan,[1] Thilagavathi Ramamoorthy[1], Krishnan Sathishkumar[1], Rohith Mohan[1], Nifty Tomy[1], Miller G J[1], Prashant Mathur[*1].

Affiliations:

[1] Indian Council of Medical Research (ICMR) – National Centre for Disease Informatics and Research (NCDIR), Bengaluru, Karnataka, India.

Corresponding Author:

Dr. Prashant Mathur, Director, ICMR – NCDIR, Nirmal Bhawan – ICMR Complex (II Floor), Poojanahalli, Kannamangala Post, Bengaluru – 562 110 (India).

Telephone: 080-22176300; Email id: diresctor-ncdir@icmr.gov.in

ORCiD ID:0000-0002-9271-1373

Supplementary Table 1. Estimated age wise distribution of female population in India at all 28 PBCRs for 2012-16.

Age group	Females
0-4	18720644
5-9	19272096
10-14	20519530
15-19	21369879
20-24	25140480
25-29	25088754
30-34	21244445
35-39	19647303
40-44	16798654
45-49	14669153
50-54	11587717
55-59	9337927
60-64	8238751
65-69	5521566
70-74	3975354
75-79	2341554
80-84	1517685
85+	1360195
**Total**	**246351687**

Supplementary Table 2. Life expectancy of females by age group as per WHO

Age group	Females
0-4	90.0
5-9	85.0
10-14	80.0
15-19	75.0
20-24	70.0
25-29	65.0
30-34	60.0
35-39	55.0
40-44	50.0
45-49	46.0
50-54	40.0
55-59	36.0
60-64	31.0
65-69	26.0
70-74	21.0
75-79	16.0
80-84	11.0
85+	5.0
